# Retinal aging in the diurnal Chilean rodent (*Octodon degus*): histological, ultrastructural and neurochemical alterations of the vertical information processing pathway

**DOI:** 10.3389/fncel.2015.00126

**Published:** 2015-04-21

**Authors:** Krisztina Szabadfi, Cristina Estrada, Emiliano Fernandez-Villalba, Ernesto Tarragon, Gyorgy Setalo Jr., Virginia Izura, Dora Reglodi, Andrea Tamas, Robert Gabriel, Maria Trinidad Herrero

**Affiliations:** ^1^Department of Experimental Zoology and Neurobiology, University of PecsPecs, Hungary; ^2^Janos Szentagothai Research CenterPecs, Hungary; ^3^Clinical and Experimental Neuroscience (NiCE), CIBERNED and Institute of Bio-Health Research of Murcia (IMIB), School of Medicine, Campus Mare Nostrum, University of MurciaMurcia, Spain; ^4^Department of Medical Biology, University of PecsPecs, Hungary; ^5^Department of Anatomy, MTA-PTE “Lendulet” PACAP Research Team, University of PecsPecs, Hungary

**Keywords:** *Octodon degus*, aging, retina, vertical pathway, ultrastructure, rod bipolar cells, synaptic proteins

## Abstract

The retina is sensitive to age-dependent degeneration. To find suitable animal models to understand and map this process has particular importance. The degu (*Octodon degus*) is a diurnal rodent with dichromatic color vision. Its retinal structure is similar to that in humans in many respects, therefore, it is well suited to study retinal aging. Histological, cell type-specific and ultrastructural alterations were examined in 6-, 12- and 36-months old degus. The characteristic layers of the retina were present at all ages, but slightly loosened tissue structure could be observed in 36-month-old animals both at light and electron microscopic levels. Elevated Glial fibrillary acidic protein (GFAP) expression was observed in Müller glial cells in aging retinas. The number of rod bipolar cells and the ganglion cells was reduced in the aging specimens, while that of cone bipolar cells remained unchanged. Other age-related differences were detected at ultrastructural level: alteration of the retinal pigment epithelium and degenerated photoreceptor cells were evident. Ribbon synapses were sparse and often differed in morphology from those in the young animals. These results support our hypothesis that (i) the rod pathway seems to be more sensitive than the cone pathway to age-related cell loss; (ii) structural changes in the basement membrane of pigment epithelial cells can be one of the early signs of degenerative processes; (iii) the loss of synaptic proteins especially from those of the ribbon synapses are characteristic; and (iv) the degu retina may be a suitable model for studying retinal aging.

## Introduction

The vertebrate retina, like other parts of the central nervous system, is subjected to degenerative changes caused by aging. The retina is also the site of diseases for which age is a major risk factor, including macular degeneration and glaucoma (Jackson and Owsley, 2003). The retina is arguably the best understood part of the vertebrate central nervous system with regard to its cellular patterning, circuitry, and function. It is composed of five major neuron types: photoreceptors, interneurons (horizontal, bipolar, and amacrine cells), and retinal ganglion cells (RGCs) that integrate visual information and send it to the brain (Sanes and Zipursky, 2010). Retinal neurons can be further subdivided into approximately 70 distinct functional subtypes (Masland, 2001) for many of which markers are available to identify the aging-specific alterations.

Age-related complications have been demonstrated in several mammalian species, including monkeys, cats, sheep, rats, mice and *Octodon degus* (degu). This latter species presents several advantages for studying different pathological conditions. The degu is a diurnal, highly visual South American hystricomorph rodent native to Chile, which in old age expresses cognitive deficits, anxiety (Popović et al., 2009) and unstable circadian rhythms of low amplitude (Vivanco et al., 2007). Particularly notable is that the animals develop spontaneous Alzheimer-like pathology and show signs of significant white matter disruption, diabetes and cancer in aging (Inestrosa et al., 2005; Ardiles et al., 2012, 2013), resembling several aspects of pathological human aging (van Groen et al., 2011).

The visual system of degus is also comparable to the human visual system. It shows robust responses to both photic and non-photic circadian Zeitgebers (Goel et al., 1999; Jacobs et al., 2003). Degus have the potential for dichromatic color vision on the basis of green-sensitive M cones and UV-sensitive (in the near UV) S cones, the most common type of mammalian color vision (Jacobs, 1993; Chávez et al., 2003; Palacios-Muñoz et al., 2014). In degus, the retinal projection is primarily contralateral, with a small ipsilateral component (Fite and Janusonis, 2001). Degu is also well suited model for studying eye pathology, because they have an increased susceptibility to cataract development and aging (Worgul and Rothstein, 1975; Brown and Donnelly, 2001; Peichl et al., 2005). However, no data are available on retinal aging in degu. In other rodents (rats and mice), retinas show some aging alterations. For example, the total retinal area expands while RGC dendritic arbors shrink with age, thus, each RGC covers a decreased fraction of the visual field in old animals. Amacrine and bipolar cells also exhibit age-related structural changes, some of which may contribute to reduced visual function (Samuel et al., 2011). Neuronal loss with age is characteristic for some, but not all species, leading to thinning of the cellular and synaptic layers (Miller et al., 1984; Limaye and Mahmood, 1987; Morrison et al., 1990; Gao and Hollyfield, 1992; Kim et al., 1996; Samuel et al., 2011). Ultrastructural changes have also been revealed in the neural, vascular and epithelial components. Even more prominent changes can be observed in the retinal pigment epithelial (RPE) layer than in the neuroretina during the early phases of aging. Signs include increased number of basal infoldings, phagolysosomes and lipofuscin deposits. In aged rat retina, organelle atrophy and whirling extensions of the basal membrane into the cytoplasm are characteristic in the RPE cells (DiLoreto et al., 2006). However, it is not known at present if these changes are also characteristic to degus. In spite of the similarities between human and degu retinas (Cuenca et al., 2010), surprisingly little is known about the degu retina and its retinal aging.

Therefore, the aim of the present study was to perform a complex retinal characterization of degu at histological, ultrastructural and immunohistochemical levels during aging focused on the elements of the vertical pathway (photoreceptors to bipolar to ganglion cells). Since degu retina is more similar to the human retina than to the retina of other rodents, this description will provide a strong foundation for future studies where experimental manipulations and/or neuroprotective agents can be studied.

## Materials and Methods

### Animals

A total of 28 female degus (body weight 180–270 g) of 6 (*n* = 8), 12 (*n* = 8), and 36-months of age (*n* = 12) were used. This latter group is considered as aging (but not old) group. Degus were housed individually in opaque glass cages (40 × 25 × 25 cm) at the animal facilities of the University of Murcia. Throughout the study, the experimental room was maintained under controlled temperature (21 ± 1°C) and 12 h light/dark cycle (lights on at 7:00 a.m. and off at 19:00 p.m.). The floors of the cages were covered with wood shavings that were changed once a week. Food and water were provided *ad libitum* by placing 120 g food pellets (Harlan Tekland Global Diet®, Harlan Laboratories, USA) per day and water bottles on a grid located on the top of the tank. The water in the tank was changed daily. All experiments were performed in accordance with relevant regulatory standards, experimental guidelines and procedures complied with the European Community Council Directive (2010/63/UE) and the ethical committee of the University of Murcia.

### Histological and Electron Microscopic Analysis

Animals were anesthetized with Isofluorane (Isoba® vet, USA), administered with a continuous flow vaporizer (MSS3, Medical Supplies and Services, England, UK), and then sacrificed by decapitation. Both eyes were immediately removed and distinctly disposed according to histological or electron microscopic procedure to be performed.

For histology eyes were fixed in 4% paraformaldehyde (PFA; Merck, Hungary) dissolved in 0.1M phosphate buffer (PB; Spektrum3D, Hungary). The eyecups were dissected and embedded in epoxy resin (Durcupan ACM resin; Sigma-Aldrich, Hungary) as we previously described (Szabadfi et al., 2012). Sections were cut at 2 μm, stained with toluidine blue (Sigma-Aldrich, Hungary), and examined in a Nikon Eclipse 80i microscope. Measurements were taken with the SPOT Basic program. Central retinal areas within 1 and 2 mm from the optic disc were used for measurements (*n* = 2–5 measurements from one tissue block). The following parameters were measured: (i) cross-section of the retina from the outer limiting membrane (OLM) to the inner limiting membrane (ILM); (ii) the width of individual retinal layers. Statistical comparisons were made using one-way ANOVA test followed by Tukey-B posthoc analysis. Data were presented as mean ± SEM (GraphPadPrism5.0).

Electron microscopy was performed on eyes fixed with 4% PFA supplemented with 1% glutaraldehyde dissolved in 0.1M PB. After washing in PB, tissue samples were treated with 1% OsO_4_ in PB, dehydrated through ascending ethanol series and embedded in Durcupan ACM resin (Sigma-Aldrich, Hungary). Sections were cut at 70 nm in Reichert Ultracut S and counterstained with Reynold’s lead citrate. Samples were examined and photographed in a JEOL 1200EX electron microscope.

### Immunohistochemistry

Eyes were dissected immediately after sacrifice in ice-cold phosphate buffer with saline (PBS) and fixed in 4% PFA at room temperature. Tissues were then washed in PBS and cryoprotected in 20% sucrose at 4°C. For cryostat sectioning, retinas were embedded in tissue-freezing medium (Shandon Cryomatrix, USA), cut in a cryostat (Leica, Germany) at 10 μm radially. Sections were mounted on subbed slides. Primary antibodies and peanut agglutinin-conjugated with FITC (PNA) were used overnight at room temperature (Table 1). Next day the sections were incubated for 2 h at room temperature with the corresponding secondary fluorescent antibodies in the dark, then coverslipped using Fluoromount-G (Southern Biotech, USA). For the colocalization study, we used 10 μm cryostat sections simultaneously with antibodies to Chx10 and protein kinase Cα (PKCα); C-terminal Binding Protein 2 (CtBP2) and Bassoon; PKCα and Bassoon; PKCα and postsynaptic density 95 protein (PSD95/SAP90); and PKCα and vesicular glutamate transporter 1 (VGLUT1), respectively. These were detected with corresponding secondary antibodies (Table 1); nuclei were counterstained with DAPI (4′, 6-diamidino-2-phenylindole; 1:10000), then coverslipped using Fluoromount-G (Southern Biotech, USA). For control experiments, primary antibodies were omitted, and cross-reactivity of the non-corresponding secondary antibodies with the primaries was also checked. Photographs were taken with Nikon Eclipse 80i Microscope (Nikon, Japan) and Fluoview FV-1000 Laser Confocal Scanning Microscope (Olympus, Japan) and further processed with Adobe Photoshop 7.0 program. Images were adjusted for contrast only, aligned, arranged, and labeled using the functions of the above program.

**Table 1:** **Antibodies used in immunohistochemical experiments**.

Primary antibodies	Company	Raised in	Dilution	Secondary antibodies	Company	Dilution
Anti-Brn3a	Santa Cruz, Hungary	Mouse	1:50	Alexa Fluor 488	Invitrogen, USA	1:1000
Anti-GFAP	Sigma-Aldrich, Hungary	Rabbit	1:500	Alexa Fluor 568	Invitrogen, USA	1:1000
PNA	Vector Labs, USA	-	1:500	-	-	-
Anti-CtBP2	BD Transduction, USA	Mouse	1:20000	Alexa Fluor 488	Invitrogen, USA	1:1000
Anti-Bassoon	AbCam, Hungary	Rabbit	1:500	Alexa Fluor 568	Invitrogen, USA	1:1000
Anti-Chx10	Thermo Scientific, Hungary	Sheep	1:100	Alexa Fluor 488	Life Technologies, Hungary	1:1000
Anti-VGLUT1	AbCam, Hungary	Rabbit	1:500	Alexa Fluor 568	Invitrogen, USA	1:1000
Anti-PKCα	Sigma-Aldrich, Hungary	Mouse	1:250	Cy 3.5	AbCam, Hungary	1:1000
Alexa Fluor 488	Invitrogen, USA	1:1000

*Abbreviations: CtBP2—C-terminal Binding Protein 2; GFAP—glial fibrillary acidic protein; PKCα—protein kinase Cα; PNA—peanut agglutinin-conjugated with FITC; VGLUT1—vesicular glutamate transporter 1*.

The number of RGCs (Brn3a-positive cells; Xiang et al., 1995; Nadal-Nicolás et al., 2009) ± SEM was measured in 100 μm ganglion cell layer (GCL) length. The cells expressing both Chx10 and PKCα were scored as rod bipolar cells, and cells expressing Chx10 but not PKCα were scored as cone bipolar cells as followed the protocol of Morrow et al. (2008). The number of all bipolar cells and rod bipolar and cone bipolar cells were counted in 100 μm^2^ area of INL. Statistical comparisons were made using one-way ANOVA test followed by Tukey-B posthoc analysis. Data were presented as mean ± SEM (GraphPadPrism5.0).

## Results

The baseline characterization of 6-, 12- and 36-month-old degu retinas was initially done by routine histology.

### Descriptive Morphological and Morphometric Analysis

**Figure 1:**
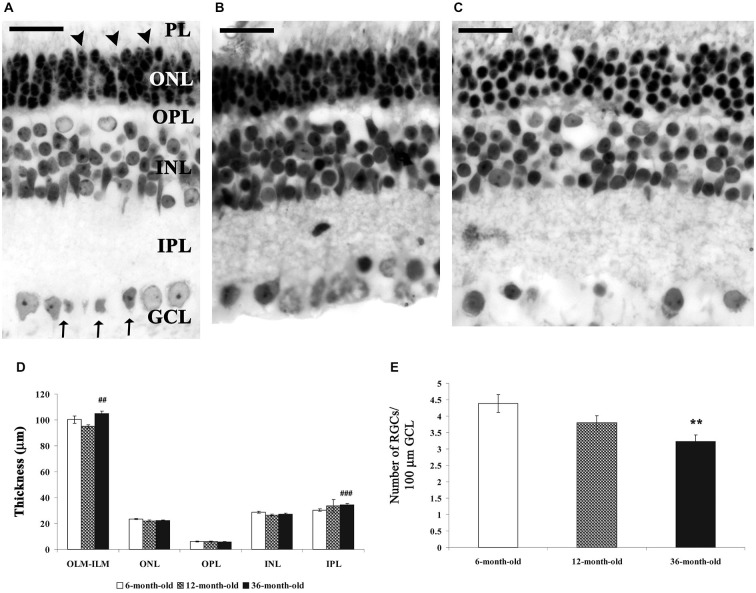
**Morphological and morphometric analysis of the 6-, 12- and 36-month-old representative degu retina sections stained with toluidin-blue**. The characteristic layers of the mammalian retina were well visible in all groups, major morphological differences could not be observed between the three groups **(A–C)**. However, the thickness of inner plexiform layer (IPL) **(D)** and the whole retina (OLM-inner limiting membrane (ILM), **(D)** significantly increased, while the RGCs (Brn3a-positive cells) number in 100 μm ganglion cell layer (GCL) length significantly decreased in the 36-month-old degus retinas compared to the 6- and 12-month-old animals **(E)**. Data are presented as mean ± SEM. **p < 0.001 vs. 6- and 12-month-old; ^##^*p* < 0.001 vs. 12-month-old; ^###^*p* < 0.0001 vs. 6-month-old degu retinas. Abbreviations: photoreceptor layer (PL); outer nuclear layer (ONL)—outer nuclear layer (ONL); outer plexiform layer (OPL)—outer plexiform layer (OPL); INL—inner nuclear layer; IPL—inner plexiform layer (IPL); GCL—ganglion cell layer (GCL); OLM—outer limiting membrane (OLM) (arrowheads); ILM—ILM (arrows); RGCs—retinal ganglion cells. Scale bar: 20 μm.

The characteristic layers of the mammalian retina were well distinguishable in degu: photoreceptor layer (PL), outer nuclear layer (ONL), outer plexiform layer (OPL), inner nuclear layer (INL), inner plexiform layer (IPL) and GCL (GCL; Figure 1). The typical cells of the mammalian retina (photoreceptor cell bodies and outer segments (OS) of cones and rods, bipolar cells, different types of amacrine cells, horizontal cells, displaced amacrine cells, ganglion cells and Müller glial cells) were also well visible at all ages.

There were only minor differences between the three groups (Figures 1A–C). A loose retinal structure could be observed in aging degu retinas (Figure 1C). This observation was manifested in a significantly increased OLM–ILM distance and IPL thickness in 36-month-old degu retinas (Figure 1D). However, the thickness of other layers did not change significantly in the aging retinas (Figure 1D; ^##^*p* < 0.001 vs. 6-month-old, ^###^*p* < 0.001 vs. 12-month-old degu retinas). The number of RGCs (Brn3a-positive cells)/100 μm retina length was significantly decreased in the 36-month-old degu retina (**p < 0.001 vs. 6- and 12-month-old degu retinas; Figure 1E) compared to the 6- and 12-month-old retinas.

### Glial Cells and The Structure of Outer Retina

**Figure 2:**
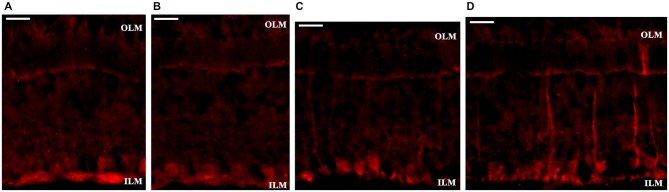
**Glial fibrillary acidic protein (GFAP) labeling of Müller glial cells**. GFAP immunostaining was observed in the endfeet of Müller glial cells in 6- and 12-month-old degu retinas **(A,B)**. In the 36-month-old degu retinas the GFAP immunoreactivity is very strong within their endfeet at the vitreoretinal border. Other processes of Müller cells in some places span from the ILM to the OLM **(D)**, but not in all retinal locations **(C)**. Abbreviations: GFAP—Glial fibrillary acidic protein (GFAP); OLM—outer limiting membrane; ILM—inner limiting membrane. Scale bar: 20 μm.

Glial fibrillary acidic protein (GFAP)-positivity was selectively localized to endfeet of Müller cells (Figures 2A–D). Müller glial cells respond rapidly to any alterations of the retinal microenvironment by elevated expression of GFAP as a specific metabolic stress signal in the mammalian retina (Cuenca et al., 2014). Increased GFAP immunoreactivity was observed in the entire width of the 36-month-old degu retina in few, but not all, Müller glial cells (Figures 2C,D), compared to 6- (Figure 2A) and 12-month-old (Figure 2B) degu retinas.

**Figure 3:**
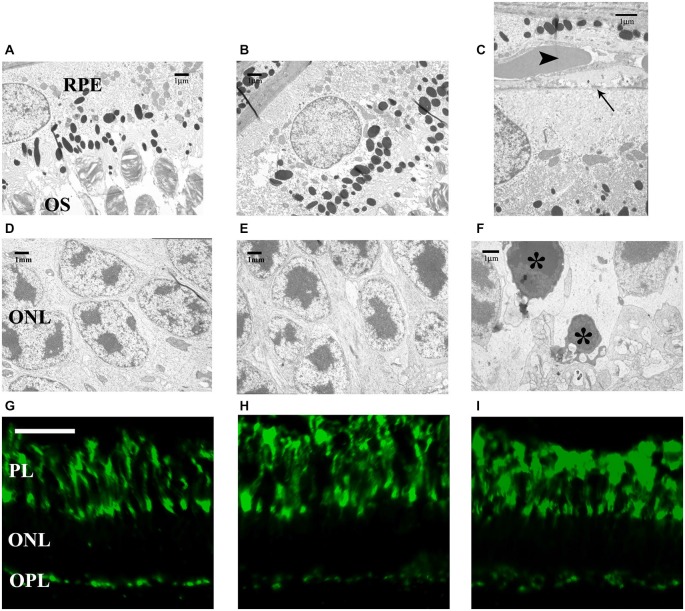
**Electron microphotographs of pigment epithelial cells, their connection to the outer segments (OS) (A–C) and somas of photoreceptors (D–F)**. Labeling of cone photoreceptor OS and terminals **(G–I)** in 6-, 12- and 36-month-old degu retinas. Ultrastructure of a retinal pigment epithelial (RPE) cell is visible above the photoreceptors OS **(A–C)**, however, the somas of the RPE cells are compressed against the basement membrane (arrow) in the 36-month-old retinas (arrowhead: blood vessel; **C**). The somas of photoreceptors seem intact in all groups **(D–F)** but some degenerative photoreceptor cell bodies and terminals appear in the 36-month-old degu retinas (asterisks; **F**). Both the cone OS and terminals show PNA in 6-month-old degu retinas, no differences could be observed in the OS and number of cone photoreceptor terminals between the three groups **(G–I)**. Abbreviations: RPE—retinal pigment epithelium; OS—outer segments (OS) of photoreceptors; ONL—outer nuclear layer; PL—photoreceptor layer; PNA—peanut agglutinin conjugated with FITC; OPL—outer plexiform layer. Scale bars are 20 μm in **(G–I)** and scale bars are indicated in all electron microscopic images **(A–F)**.

Basement membrane proliferation of the RPE in the areas of age-related retinal peripheral degeneration was observed in the 36-month-old degus retinas. The thickening of Bruch’s membrane and fibrosis of the choriocapillary were evident. The somas of the RPE cells were pressed toward this thinner basement membrane in the 36-month-old retinas, suggesting an initial age-related alteration of the RPE. Early peripheral changes of the RPE included increased basal infoldings, phagolysosomes and lipofuscin deposits, as well as atrophy and whirling extensions of the basement membrane into the cytoplasm (Figure 3C). The relation between RPE and the OS of the photoreceptors seemed intact in all groups (Figures 3A–C).

We observed that the cones composed a nest between the rods in all age groups, in accordance with the cone-dominant retinal structure of the degu (Cuenca et al., 2010). Most of these cone-nests were located near the OLM (Figures 3D–F). In the 36-month-old group, we detected altered photoreceptor ratio for the favor of cones suggesting that rods are more sensitive to aging. We also observed degenerating rods in the ONL of the 36-month-old group (Figure 3F). PNA is used to label the OS and the terminals of cone photoreceptors in the OPL. No differences could be observed in the OSs between the three different age groups (Figures 3G–I) and the number of cone terminals (data not shown), which further supports the selective loss of rods at 36-months.

**Figure 4:**
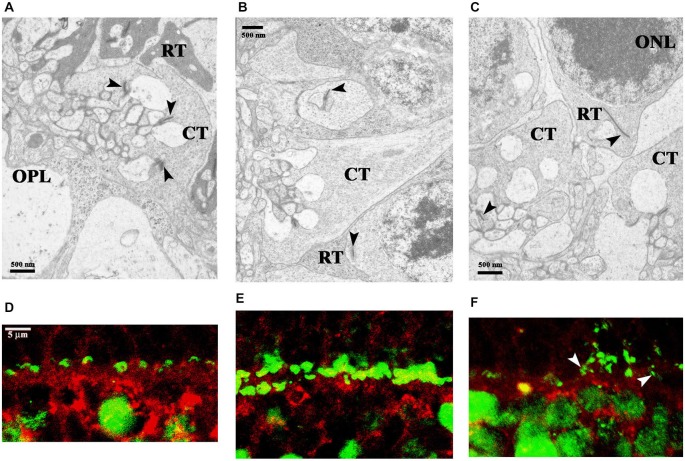
**Electron microphotographs of representative retinal locations in OPL (A–C) and ribbon synaptic markers in the OPL (D–F)**. Ribbon synapses (arrowheads) in the OPL. At the fine structural level, the major difference between the three groups is the reduced cytoplasmic density of the rod terminals (RT) in aging animals **(A–C)**. The synaptic profiles marked with horseshoe-shaped rod ribbons by CtBP2 (green) and Bassoon (red) labeling **(D–F)**. Near the horseshoe-shaped ribbons, fragmented ribbons (white arrowheads) could also be observed in 36-month-old degu retinas **(F)**. Abbreviations: CT—cone terminals; ONL—outer nuclear layer; OPL—outer plexiform layer; RT—rod terminals; CtBP2—C-terminal Binding Protein 2. Scale bars are indicated in the images **(A–C)** and 5 μm in **(D–F)**.

We found ribbon synapses in the OPL and observed differences between the rod and the cone terminals. The rod terminals (RT) were more electron dense and cup-shaped spherules, while cone terminals were bowl-shaped, less dense pedicles (Figures 4A,B). The only difference between the three groups was the reduced cytoplasmic density of the RT with older age. As a consequence we could not easily distinguish the rod and the cone terminals in the 36-month-old groups (Figure 4C).

Photoreceptors transmit their signals at ribbon synapses in the OPL, the first synaptic region in the retina, whereas bipolar cells make their ribbon synaptic contacts in the IPL. A large number of regularly aligned synaptic vesicles were tethered to the ribbon. The rod photoreceptor ribbon synapses had horseshoe-shaped structure in the OPL, however, the cone photoreceptor (OPL) and bipolar cell ribbons (IPL) had a dot-like appearance at light microscopic level. We analyzed the structure of the OPL with retina-specific ribbon synapse markers presynaptic CtBP2 and Bassoon labeling. The rod ribbon synaptic profiles were marked with horseshoe-shaped ribbons by CtBP2 and continuous distribution of punctate staining by Bassoon protein (Figures 4D,E). Near the horseshoe-shaped ribbons, some degenerated synaptic structures (fragmented ribbons) could be observed in the OPL of 36-month-old degu retinas (Figure 4F). The localization of Bassoon was similar in the three groups (Figures 4D–F).

### Bipolar Cells and Ultrastructure of the Synaptic Layers

**Figure 5:**
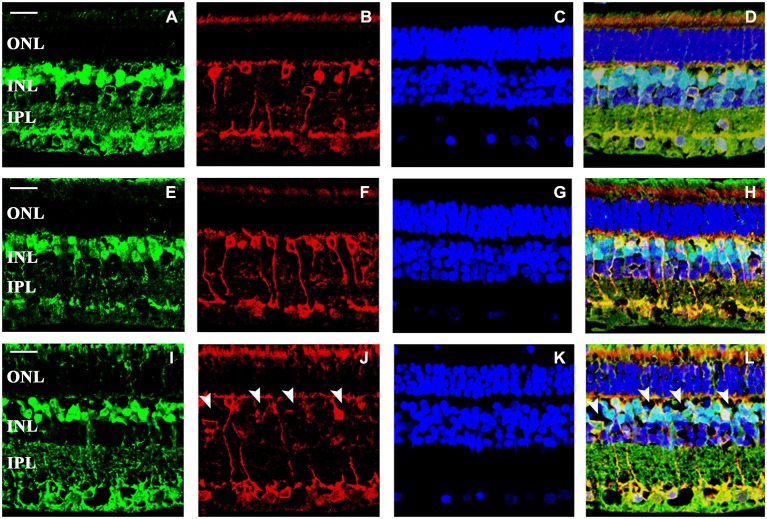
**Representative retinal sections stained with pan-bipolar and rod bipolar cell markers**. Pan-bipolar cell marker (Chx10) labeled the somas and terminals of all bipolar cells (green—**A,E,I**; light blue—**D,H,L**), while protein kinase Cα (PKCα) labeled the somas, dendrites and axon terminals of rod bipolar cells (red—**B,F,J**; yellow, white—**D,H,L**). DAPI staining labels the nuclei of all retinal cells (dark blue—**C,G,K**). The staining pattern was similar in all groups **(A–L)**, except that empty cell body shapes (arrowheads) could be observed among the bipolar cells in the 36-month-old degus retina **(I–L)**. Abbreviations: ONL—outer nuclear layer; INL—inner nuclear layer; IPL—inner plexiform layer; PKCα—protein kinase Cα (PKCα). Scale bar: 20 μm.

The pan-bipolar cell marker Chx10 reveals the organization of bipolar cells in the INL of the retina (Elshatory et al., 2007). We found that anti-Chx10 antibodies stained all bipolar cells in all groups (Figures 5A,E,I). The presence of PKCα was detected in the rod bipolar cell population. The labeled structures were the cell bodies of the INL, the dendrites in the OPL and cell processes extending into the IPL, close to the GCL. There were no alterations detected in the arborization and pattern of the cone and rod bipolar cells between the 6- and 12-month-old groups (Figures 5A–L). In the 36-month-old retinas empty cell body-like shapes could be observed in the bipolar cell area of INL (Figures 5I,J,L).

**Figure 6:**
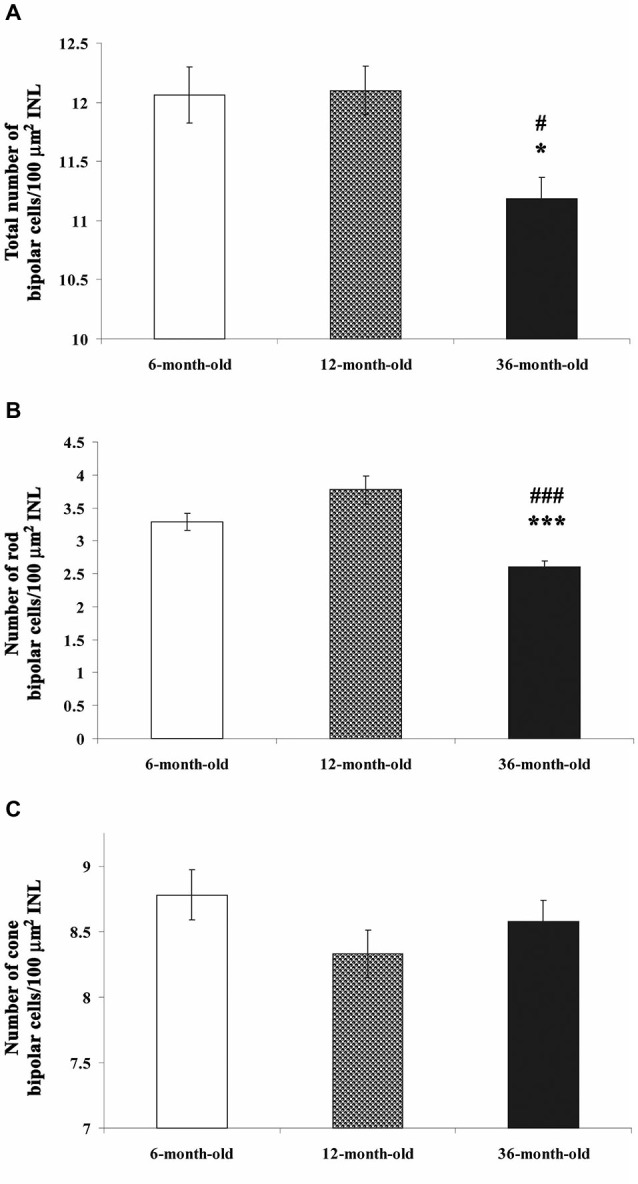
**Quantification of all bipolar cells (A), only rod (B) and cone bipolar cells (C) in each group**. The cell number of all bipolar cells and rod bipolar cells/100 μm^2^ INL was significantly decreased in 36-month-old degu retinas (**A,B**; *p < 0.05; ***p < 0.0001 vs. 6-month-old; ^#^*p* < 0.05; ^###^*p* < 0.0001 vs. 12-month-old degu retinas). No differences could be observed in the cone bipolar cell number between the three groups **(C)**.

Differences could be observed in the total bipolar and rod bipolar cell numbers (*p < 0.05 vs. 6- and ^#^*p* < 0.05 vs. 12-month-old groups and ***p < 0.0001 vs. 6- and ^###^*p* < 0.0001 vs. 12-month-old groups, respectively). The most prominent difference was at the age of 36 months with a significant reduction in these parameters (Figures 6A,B). However, the number of cone bipolar cells did not differ between the three groups (Figure 6C). These alterations resulted in an increased cone/rod bipolar cell ratio in the 36-month-old degu retina.

**Figure 7:**
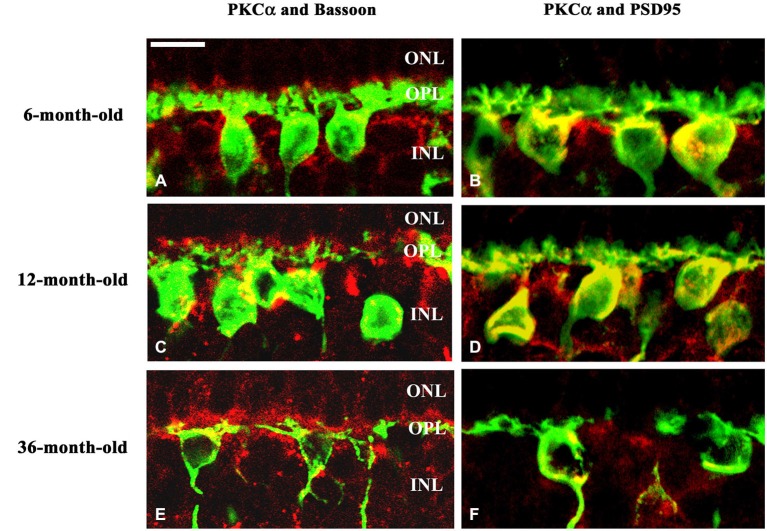
**Spatial relationship of the dendritic arbor of rod bipolar cells and photoreceptor terminals**. Double immunostaining with PKCα (green) and Bassoon (red) antibodies **(A,C,E)**. The dendrites of rod bipolar cells were longer and more brushy in the 6- and 12-month-old degu retinas **(A,C)**, than in the 36-month-old retinas **(E)**. Double immunolabeling with PKCα (green) and PSD95 (red) antibodies shows the related alterations in the rod synaptic spherules and in the dendritic field of rod bipolar cells. PSD95 immunofluorescence is prominent in the OPL, where the axon terminals of rods, the so-called rod spherules, are labeled intensively in the 6- and 12-month-old degus retinas **(B,D)**. Decreased PSD95 labeling in the reduced dendritic arbors of rod bipolar cells was shown in 36-month-old aging degu retina **(F)**. Abbreviations: ONL—outer nuclear layer; OPL—outer plexiform layer; INL—inner nuclear layer; PKCα—protein kinase Cα; PSD95—postsynaptic density 95 protein. Scale bar: 10 μm.

The dendritic field of rod bipolar cells was also altered. The progression of the degeneration was manifested by the retraction of rod bipolar cell dendrites. These dendrites became flatter and the loss of dendritic branches above the cell bodies was evident. In the 36-month-old group the PKCα positive dendrites were sparsely distributed (Figures 7E,F) compared to the 6- and 12-month-old groups. The pattern of PKCα positive dendrites was dense and each cell had a huge arbor in these latter two groups (Figures 7A–D). In normal retina the fine processes of rod bipolar cells penetrate the ONL. The dendritic trees appear brushy and candelabrum-like as we showed in the case of 6-month-old degus (Figures 7A,B). In 36-month-old degu retinas the rod bipolar cell dendrites were no longer erect and brushy, but appeared flattened (Figures 7C,F). The Bassoon-staining seemed to be unchanged during aging (Figures 7A,C,E). Glutamatergic photoreceptors affect the physiological properties of bipolar cells in the mammalian retina. To visualize the spatial pattern of glutamatergic input to the bipolar cells in the OPL of degu retina, PSD95 was used to label these synapses. PSD95 puncta were regularly spaced along the membranes of the bipolar cell dendrites. The spatial distribution of PSD95 labeling of the bipolar cells were similar in the 6- and 12-month-old groups (Figures 7B,D). However, in the 36-month-old group the pattern of PSD95 was altered, only a few puncta were detected on the somas of rod bipolar cells and labeling was not shown along the non-brushy dendritic arbor (Figure 7F).

**Figure 8:**
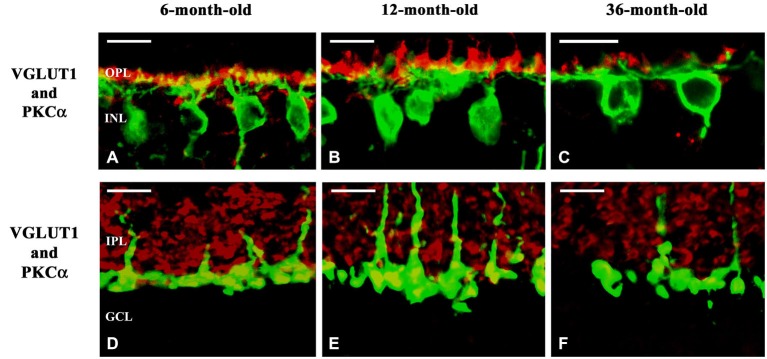
**Confocal microscopy of degu retinal sections immunolabeled with VGLUT1 (red) and PKCα (green)**. Subcellular localization of VGLUT1 in the outer **(A–C)** and inner plexiform layers **(D–F)** of 6- **(A,D)**, 12- **(B,E)** and 36-month-old **(C,F)** degu retinas. VGLUT1-immunopositive structures were found in photoreceptor terminals in close apposition to PKCα-positive rod bipolar cell dendrites in the OPL of 6- and 12-month-old degu retinas **(A,B)**. Altogether with the decreased dendritic arbor of the rod bipolar cells the VGLUT1-expression in photoreceptors also decreased in 36-month-old degu retinas **(C)**. VGLUT1-positive large puncta were co-localized with PKCα in the inner part of the IPL indicating VGLUT1 expression in axon terminals of rod bipolar cell in 6- and 12-month-old degu retinas **(D,E)**. In the 36-month-old degu retinas both the arbor of axon terminals of rod bipolar cells and their VGLUT1 expression decreased **(F)**. Abbreviations: OPL—outer plexiform layer; INL—inner nuclear layer; IPL—inner plexiform layer; GCL—ganglion cell layer; PKCα—protein kinase Cα; VGLUT1—vesicular glutamate transporter 1. Scale bar: 10 μm.

VGLUT1 was detected in the OPL and throughout the laminae of the IPL, a distribution consistent with the expected synaptic localization of the protein (Brandstätter et al., 1999). In degus, the coarse structure of the retina was not significantly affected, however, the axon terminals of rods and rod bipolar cells showed dramatic alterations in VGLUT1 expression in the 36-month-old group (Figures 8C,F). These degu retinas showed loss of most of the rod outputs to their bipolars (Figures 8C,F) compared to 6- (Figures 8A,D) and 12-month-old (Figures 8B,E) degu retinas. The axon terminals of the rod bipolar cells showed some alterations in the 36-month-old group, such as decreased staining both for PKCα and VGLUT1 in the innermost IPL. The loss of PKCα immunoreactivity in the axons and axon terminals of rod bipolar cells was also evident and their terminals were further reduced in size and density (Figure 8F) compared to 6- (Figure 8D) and 12-month-old (Figure 8E) retinas.

**Figure 9:**
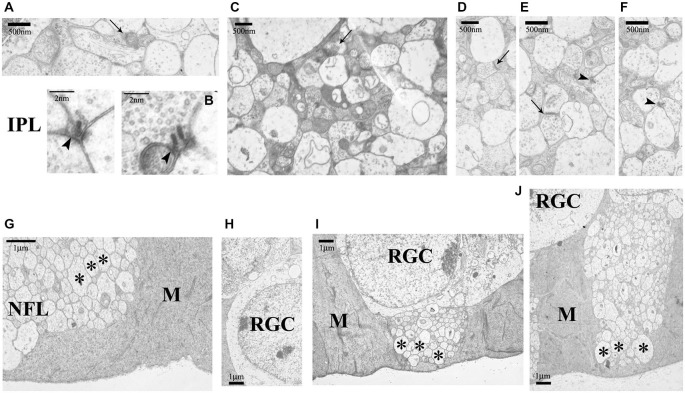
**Ultrastructure of IPL, GCL and nerve fiber layer (NFL) in the 6-, 12- and 36-month-old degu retinas**. In the IPL conventional (arrows) and ribbon synapses (arrowheads) were also present in all groups (6-month-old: **(A,B)**; 12-month-old: **(C)**; 36-month-old: **(D–F)**). The NFL is intact in all age groups (6-month-old: **(G,H)**; 12-month-old: **(I)**; 36-month-old: **(J)**; asterisks: nerve fibers). Abbreviations: IPL—inner plexiform layer; M—Müller glial cell; NFL—nerve fiber layer (NFL); RGC—retinal ganglion cell. Scale bars are indicated in the pictures.

The IPL structure was well retained (Figures 9A–F) even at 36 months of age (Figures 9D–F). Both ribbon and conventional synapses were visible, synaptic vesicles were regularly distributed. Sometimes a few swollen neural profiles (lacking synaptic vesicles) were seen along with space-filling glial protrusions which can be clearly identified in the electron microscope. The structural elements of the GCL, nerve fiber layer (NFL) and ILM in general did not show major signs of degeneration in any of the groups (Figures 9G–J). The NFL fibers were embedded into the large endfeet of Müller glial cells (Figures 9G,I,J).

## Discussion

Progressive and irreversible functional decay during aging is characterized by region-specific neuron loss (Lossi et al., 2005). The retina is a potentially sensitive target of age-dependent degeneration. In this paper, we report a comparison of the major retinal neuronal types of the vertical pathway in young adult (6-months-old), adult (12-months-old) and aging (36-months-old) degus. There are several remarkable differences along with numerous unchanged features in the different age groups.

**Figure 10:**
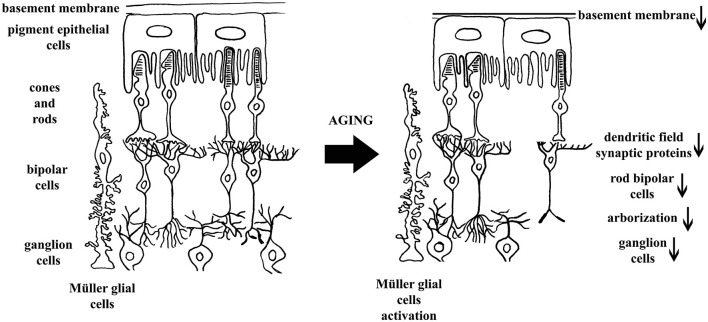
**Schematic representation of concluded structural and cellular changes during retinal aging in 36-month-old degu retina**. In the left panel major structural elements of the vertical processing pathway are indicated while in the right panel the alterations caused by aging retina are emphasized. The direction of changes is indicated with arrows.

In our study we focused on the elements of the vertical information processing pathway with the addition of the RPE and the Müller glial cells. In the retinas of 36-month-old animals we observed a slightly loosened tissue structure both at light and electron microscopic levels, elevated GFAP expression in Müller glial cells and reduced number of rod bipolar cells and RGCs. Other age-related differences were detected at ultrastructural level: alteration of the retinal RPE and degenerated photoreceptor cells, especially rods, was evident. Ribbon synapses in the OPL were sparse and often fragmented (Figure 10).

We revealed well-defined alterations in degus that are also characteristic in human, mouse and rat retinal aging. The neuroretina together with RPE cells form a functional unit of the visual system. The RPE usually bears very long sheet-like apical microvilli that project into a complex matrix. During aging the RPE undergoes a number of well characterized changes, including increase in the number of residual bodies and accumulation of basal deposits (Garron, 1963; Guymer et al., 1999). We observed degeneration of the RPE/Bruch’s/choriocapillary complex in 36-month-old degu retinas, possibly leading to altered tissue oxygen levels and contributing to photoreceptor cell loss (DiLoreto et al., 2006). The degeneration process is complex and involves the accumulation of deposits, RPE cell loss leads to formation of hypopigmented areas, and the development of hyperpigmented areas. This stage can progress to a proliferative neovascular (wet or exudative) form of degeneration characterized by the growth of choroidal vessels (choroidal neovascularization) or to a geographic form of atrophy characterized by damage of the RPE and of the neural retina (Limaye and Mahmood, 1987). Some signs of this process could already be seen at 36- months of age and we predict that these changes will become dominant at later ages. The observed elevated cone/rod bipolar cell ratio in the 36-month-old degu group indicates that the elements of cone pathway were more resistant to the age-related degeneration than that of rods. This parallels well with the observation that in the human retinas there is a decrease of approximately 54% of the total rod photoreceptor density between the fourth and ninth decades of life (Gao and Hollyfield, 1992; Aggarwal et al., 2007) whereas cone density remains essentially unchanged (Curcio et al., 1993). The decrease in rod density ultimately triggered the associated decline of neurons connected to them (Gao and Hollyfield, 1992; Aggarwal et al., 2007).

Photoreceptors transfer the visual signals to the post-receptorial retinal network; malfunctioning of this process due to degeneration of rods, rod bipolar cells, or ribbon synapses will lead to impaired vision. During human rod degeneration, surviving rods, horizontal and amacrine cells similarly extend anomalous neurits throughout the retina (Li et al., 1995; Fariss et al., 2000). Photoreceptor degeneration-dependent modifications in the synaptic machinery connecting photoreceptors with second-order neurons are evident: altered connectivity of rods and rod bipolar cells as well as horizontal cells affects retinal circuity. The normal pairing of presynaptic and postsynaptic markers are lost. The synaptic markers associated with photoreceptors and processes of bipolar and horizontal cells show abnormalities prior to significant photoreceptor loss (Cuenca et al., 2005). We report here age-dependent structural changes at the ribbon synapses in the synaptic terminals of rod photoreceptor and rod bipolar cells, which conforms well with these observations.

In contrast to the observed initial decline of ribbon synapses and rod bipolar cells density, the loss of synaptic sites was not complete in the aging degu retina, since Bassoon staining persisted marking the functional integrity of the arciform density. Currently we do not know whether the rods or the rod bipolars are lost first. In other pathological conditions, for example in a rat model of hyperoxia, the loss of bipolar dendrites takes place before photoreceptor death (Dorfman et al., 2011).

Putative structural modifications of the inner retina can be a consequence of aging. Liets et al. (2006) have indeed shown aberrant processes in rod bipolar neurons as a consequence of aging. Aberrant processes establish normally structured synapses ectopically (Terzibasi et al., 2009). As the degeneration of rod bipolar cells progresses they display early retraction and loss of dendrites (Cuenca et al., 2005). In 36-month-old degu retinas the rod bipolar cell dendrites were no longer erect and brushy but appeared flattened in contrast to the normal retina, where the fine processes of rod bipolar cells penetrate the ONL, like those in the 6-month-old degus.

The PSD95 is a part of the dense structure attached to the postsynaptic membrane opposed to the presynaptic active zone to ensure normal synaptic transmission. The structural alterations in aged learning-impaired rats correlate with altered content of PSD proteins that are critically involved in normal synaptic function. The alterations in synaptic protein content resulted in reduced synaptic function (Nyffeler et al., 2007; Takada et al., 2008). Immunofluorescence for PSD95 was most prominent in the retina, the dendrites in the OPL opposed to the rod spherules and cone pedicles were strongly labeled (Koulen et al., 1998). The spatial distribution of PSD95 labeling on the bipolar cell dendrites was altered in the 36-month-old group: only a few puncta were detected, furthermore, labeling was not shown nearby and along the non-brushy dendritic arbors of rod bipolar cells, indicative of rod degeneration. At the same time, however, cones remained unaltered as it was proven by PNA-labeling. The specialization of rod and cone bipolar cells involves the differential expression of proteins involved in glutamatergic signaling. Hanna and Calkins (2007) described that 26 rod bipolar cells expressed at least one AMPA glutamate receptor subunit gene in monkey retina. This infers the presence of those scaffolding proteins (including PSD95) that are related to the ionotropic glutamate receptors (Hanna and Calkins, 2007). It is known that ischemia induces severe progressive inner retinal degeneration and down-regulation of synaptic proteins, such as PSD95 and synaptophysin (Guo et al., 2014). Although PSD95 is a very important protein in the retinal synaptic transmission in both the OPL and IPL, there is no information on its alteration in retinal aging. The decreased PSD95 expression in aging degu retinas suggested that the synthesis of PSD95 could be altered.

VGLUT1 was localized to photoreceptor and bipolar cell terminals, which is consistent with the function of photoreceptors and bipolar cells in vertical excitatory transmission with glutamate release in mammalian retina (Gong et al., 2006). As a result of photoreceptor degeneration VGLUT1-immunostaining was decreased in the OPL. Remodeling of bipolar cells during aging also affects their axon terminals. These reduced axon terminals of the rod bipolar cells showed reduced VGLUT1-staining and the shape and structure of terminals was also altered in 36-month-old degu retinas. Response of VGLUTs to diverse stimuli is altered with aging, for example after transient global cerebral ischemia in the rat brain (Llorente et al., 2013) or ischemia and excitotoxicity in the retina (Atlasz et al., 2008, 2010). Similarly to our findings, decreased VGLUT1-immunostaining was observed in aging ventral cochlear nuclei, possibly associated with age-related hearing loss (Alvarado et al., 2014).

These above observations suggest that both the input and the output synapses of the rod bipolar cells were affected in the aging degu retina. In contrast, the cone pathway appeared mostly unchanged in the aging degu retinas. It is possible that this only reflects the different time course of the degeneration of rods and cones in the aging process. Rod bipolar cells and rods disappear first, therefore, the secondary degeneration within the rod pathway is expected to occur earlier. This observed alteration in aging has serious consequences in the light of the fact that one rod bipolar cell makes synapses with multiple RT via their dendritic arbor (Wässle and Boycott, 1991). With advancing age, the reported significant decrease of the rods coincides with considerable reduction in the density of rod bipolar cells in humans (Gao and Hollyfield, 1992; Aggarwal et al., 2007), similarly to degus. Not all retinal cells are equally vulnerable to the effects of advancing age (Roufail and Rees, 1997). Marked differences in the 36-month-old degus were partly cell type specific, such as elevated GFAP expression in the Müller glial cells, photoreceptors (especially rods) and rod bipolar cell loss with the alteration in their synaptic profile (ribbon synapse and altered dendritic trees: no longer erect and brushy). In contrast the bipolar cells of mice show arbor-specific alteration; their dendrites are sprouted but remain stable (Samuel et al., 2011).

Furthermore, the number of RGCs, the output neurons of the retina, was altered in degus. The prominent loss of RGC axons in the optic nerve as described across mammalian species must translate *ipso facto* to the corresponding decline in RGC bodies in the retina, whether RGC bodies in the retina are also susceptible to age-related loss (Calkins, 2013). The number of RGC bodies in the rat and mouse retina does not change with age though the retina itself enlarges and RGC shrink with a concomitant decrease in the density of IPL synapses (Harman et al., 2003; Samuel et al., 2011). In contrast, only the human retina appears to progress to actual RGC body loss with age; perhaps progression in normal aging depends on the actual extent of the lifetime (Calkins, 2013). Degu retina in this respect behaves like human retina, making it a suitable model for examining ganglion cell loss mechanisms. Altogether, these data reveal selective age-related alterations in the neural circuitry in the degu retina. These changes in the degu retina seem more closely related to those observed in human retinal aging than alterations observed in other rodents, such as rats and mice. Animal models of retinal aging have usually employed nocturnal species (e.g., rats and mice), however, to better approximate the human retinal changes during aging, the diurnal rodent, *Octodon degus* is more useful, since the (i) rod/cone ratio is similar to the human ratio; (ii) the diurnal behavior is charactheristic to this species and its activity pattern resembles that of the human; (iii) its lifespan is considerably longer than those of the other experimental models, therefore the age-related changes can be better monitored and compared to those described in the human retina; and (iv) other, age-related diseases (e.g., cataract, diabetes, Alzheimer’s disease, cancer) often appear spontaneously in this species (Jacobs, 1993; Brown and Donnelly, 2001; Chávez et al., 2003; Peichl et al., 2005; Ardiles et al., 2013; Palacios-Muñoz et al., 2014).

The proper functioning of the nervous system (including that of the retina) depends on the underlying structure of neural networks. Any loss of the pre- and/or postsynaptic profiles of the retinal neurons causes changes in their morphology and function. As a consequence, these retained neurons may be capable to establish new synaptic contacts (ectopic synapses, unusual synaptic arrangements), so the retina may undergo marked remodeling. In the aging degu retina, the synaptic rearrangements/alterations in the OPL and potentially concomitant synaptic alterations in the IPL efficacy would reduce transmission, while the loss of function of the RPE cells would alter the homeostasis of the retinas. The neuronal elements of the vertical pathway such as rod bipolar and ganglion cells were seen affected already at 36-month of age. We think that these structural changes become more obvious, will reach other cells and will more seriously affect the synaptic complexes with advancing age.

The proven similarities between the degus and the human retina in their structure and aging processes offer a possibility to develop potential treatments and therapies for retinal age-related alterations and diseases.

## Authors Contribution

KS researched data and wrote, edited and reviewed the manuscript; CE, EF-V, ET, VI and GS Jr. researched data; RG and MTH researched data, reviewed and edited the manuscript; DR and AT reviewed the manuscript.

## Conflict of Interest Statement

The authors declare that the research was conducted in the absence of any commercial or financial relationships that could be construed as a potential conflict of interest.
